# O.6.1-5 Potential moderators of the association between fundamental movement skills and academic attainment of adolescents: a path analysis

**DOI:** 10.1093/eurpub/ckad133.264

**Published:** 2023-09-11

**Authors:** Kelly A Mackintosh, Philip J Hill, Melitta A McNarry

**Affiliations:** Applied Sports, Technology, Exercise and Medicine (A-STEM) Research Centre,Swansea University, UK; k.mackintosh@swansea.ac.uk; Applied Sports, Technology, Exercise and Medicine (A-STEM) Research Centre,Swansea University, UK; k.mackintosh@swansea.ac.uk; Applied Sports, Technology, Exercise and Medicine (A-STEM) Research Centre,Swansea University, UK; k.mackintosh@swansea.ac.uk

## Abstract

**Purpose:**

Fundamental movement skills (FMS) provide a crucial foundation for the development of complex movement patterns involved in physical activity and sport. Further, FMS and skill acquisition are proposed to have a moderating influence on the association between physical activity and cognitive stimulation. Despite this, there remains scant literature investigating this relationship in adolescents. Therefore, the purpose of this study was to identify the association between object control and locomotor skills and subject-specific academic attainment in adolescents, and to ascertain whether this association is moderated by perceived sports competence, sex, weight status and/or maturational status.

**Methods:**

FMS were assessed in 140 children (70 girls; 12.8±1.9 years) using the Test of Gross Motor Development V.3 (TGMD-3). Numeracy reasoning, numeracy procedural and reading were determined using standardised national assessments and potential moderating variables (perceived sports competence [Children and Youth Physical self-perception profile – Youth], Body Mass Index (BMI) z-scores, and biological maturity [predicted age at peak height velocity]) were recorded. Path analysis was performed using maximum likelihood estimation to analyse the hypothesised direct and moderated associations between FMS and each aspect of academic attainment, whilst controlling for age and sex. The moderating role of sex was subsequently assessed using multi-group analysis and comparing a fully-constrained model. The moderating effects of perceived sports competence, BMI z-score and biological maturity were analysed using the interaction-moderation approach.

**Results:**

Path analysis revealed a significant association between object-control skill competence and both numeracy procedural (*β*=0.33; p = <0.001) and numeracy reasoning (*β*=0.29; p = <0.001). In contrast, there was no association between locomotor skills and any parameter of academic attainment. Perceived sports competence moderated the association between object-control skills and numeracy procedural (*β*=0.16; p = 0.045) and reading (*β*=0.25; p = 0.003), while BMI z-score moderated the association between object control skills and numeracy reasoning (*β*=0.17; p = 0.048).

**Conclusions:**

These results highlight the importance of object-control skills for cognitive development in adolescents. The continued development of FMS, specifically object-control skills, within cognitively-enriched experiences should be a key strategic aim in the curriculum. These findings are imperative for school buy-in for school-based physical activity promoting interventions.

